# NEMS generated electromechanical frequency combs

**DOI:** 10.1038/s41378-024-00860-9

**Published:** 2025-01-15

**Authors:** Sasan Rahmanian, Hamza Mouharrar, Rana Abdelrahman, Masoud Akbari, Yasser S. Shama, Kevin Musselman, David Muñoz-Rojas, Skandar Basrour, Eihab Abdel Rahman

**Affiliations:** 1 Department of Systems Design Engineering, University of Waterloo, Waterloo, ON N2L 3G1 Canada; 2 Renewable Energy Engineering Department, Mediterranean Institute of Technology, South Mediterranean University, Lac 2, 1053 Tunis, Tunisia; 3 Department of Mechanical and Mechatronics Engineering, University of Waterloo, Waterloo, ON N2L 3G1 Canada; 4 University Grenoble Alpes, CNRS, Grenoble INP, LMGP, 38000 Grenoble, France; 5 University Grenoble Alpes, CNRS, Grenoble INP, TIMA, 38000 Grenoble, France

**Keywords:** NEMS, Nanoscale devices

## Abstract

This paper presents a novel technique for low-power generation of frequency combs (FC) over a wide frequency range. It leverages modal interactions between electrical and mechanical resonators in electrostatic NEMS operating in air to provide a simple architecture for FC generators. A biased voltage signal drives the electrical resonator at resonance which is set to match an integer submultiple of twice the mechanical resonator’s resonance. Experimental results demonstrate that the NEMS displacement exhibit more than 150 equidistant peaks in the case of a 2:1 modal interaction and more than 60 equidistant peaks in the case of a 1:1 modal interaction. In both cases, the Free Spectral Range (FSR) was equal to the mechanical resonance frequency. Comparison between the FCs generated by the 2:1 and 1:1 modal interactions demonstrate the superiority of the former in terms of bandwidth and stability. The superior phase coherence of the FC generated via the 2:1 modal interaction was demonstrated via time-domain analysis. Our technique has the flexibility to generate multiple frequency combs and to fine-tune their FSR depending on the number of mechanical modes accessible to and the order of the activated modal interaction. It can be integrated into portable devices and is well aligned with modern miniaturization technology.

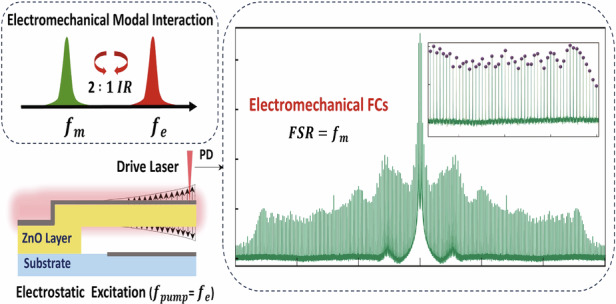

## Introduction

Optical frequency combs (OFCs) are indispensable tools with a wide range of applications in metrology, astronomy, cosmology, and atomic clocks^1–7^, and they have driven significant progress in these fields^4,8–11^. OFCs are generated through various mechanisms, each offering unique advantages and limitations. Notably, a new frontier has emerged with the advent of mechanical frequency combs (FCs), generated through coupled mechanical oscillators, resulting in side-bands with fixed phase relationships^1,10–12^. Experimental evidence confirms the presence of mechanical FCs in various systems, establishing connections with established nonlinear dynamics concepts. The consistent frequency and phase relationships within these mechanical comb teeth enable the application of frequency conversion and optical comb techniques.

Mode-locked lasers (MLLs) stand out as the predominant method for generating OFCs^2,3,13–17^. MLLs produce sequences of ultrashort optical pulses at a consistent repetition rate *f*_*r*_ with the pulse width matching comb tooth spacing. These MLLs offer remarkable stability and precision, generating high-resolution combs in the THz range. Accurate control of laser repetition rate *f*_*r*_ and offset frequency *f*_∘_ is critical for effective comb generation^2^. The methodology has evolved to include carrier-envelope phase stabilization through closed-loop feedback^13,15^, as well as the use of continuous-wave laser-pumped resonators to facilitate comb emission via cascaded third-order parametric processes (*X*^(3)^)^18^. Recent advancements have demonstrated direct generation of optical FCs in continuous-wave-pumped quadratic nonlinear resonators, involving quadratic (*X*^(2)^) processes.

Microresonators offer an alternative route for comb generation, with their compact, high-Q optical cavities supporting whispering gallery modes, resulting in comb-like spectra^3,7,19–22^. They excel in terms of size, efficiency, and stability^19–21^. Electro-optic modulation, although allowing adjustable comb characteristics, faces challenges related to stability due to microwave source phase noise and component fluctuations^8,18,23–25^. In contrast, optomechanical FCs have emerged as an innovative approach, combining mechanical oscillators and optical cavities to modulate cavity resonance^1,4,6,25–29^. This method offers advantages such as energy efficiency, reliability, and the potential for on-chip integration. Mechanical motion, driven by lasers and amplified through resonators, facilitates optical comb generation^26,27^. Adjustable comb tooth spacing has been demonstrated through amplitude modulation^26^, and modal expansion has been employed to investigate optical FCs with whispering gallery mode resonators^25^.

Phononic FCs emerge by coupling acoustic waves to optical resonators, modulating optical material refractive index, and producing optical FCs^8,10,12,30,31^. These combs offer high precision and sensitivity to external mechanical forces. Elongated microresonators with nanoscale parabolic effective radius variations, as studied by Sumetsky^25^, reveal closely spaced and dense optical eigenfrequencies linked to axially symmetric acoustic mode eigenfrequencies. De Cao et al.^12^ introduced cubic nonlinearities to generate fast and slow clocks, resulting in FCs around each eigenmode. Maksymov et al.^30^ proposed an acoustic-based mechanism for creating equidistant frequency spectra in scenarios where light-based methods are impractical.

Recent years have seen a growing interest in creating FCs by harnessing inherent nonlinearities in the dynamics of electromechanical resonators^10,32–34^. This pursuit involves orchestrating modal interactions and leveraging embedded nonlinearities. Theoretical and experimental studies have explored internal resonance (IR), such as 1:1^35–37^, 1:2^35,38,39^ and 1:3^36,40^ IR, to generate mechanical FCs. Their center frequency is the natural of a directly excited mode. The comb is obtained via a secondary Hopf bifurcation, resulting from the IR, that elicit quasi-periodic responses. Extensive experimental and theoretical investigations have realized this secondary Hopf bifurcation along a large-amplitude branch within M-shaped frequency-response curves^32^. These nonlinear phenomena facilitate efficient energy transfer between mechanical modes driven by an externally applied electrical signal.

In this work, we introduce an efficient and cost-effective paradigm for FC generation. Leveraging electromechanical modal interaction in an NEMS resonator, we achieved enhanced spacing resolution compared to complex methods. This approach ensures uniform and equally spaced combs, essential for various applications, with minimal power consumption and a compact footprint. 2:1 modal interaction outperforms the 1:1 modal interaction in terms of comb bandwidth and stability. The NEMS-based architecture seamlessly integrates into portable devices and aligns with modern miniaturization trends. This pioneering contribution caters to the growing demands of simplified, modern technology in the FC generation field.

## Results and discussion

### Device and characterization

The device-under-test (DUT) is a nano cantilever beam made of a 200 nm-thick Zinc Oxide (ZnO) layer that serves as both a structural and a functional material, unlike the case for conventional sensors. The DUT is fabricated using Atmospheric Pressure Spatial Atomic Layer Deposition (AP-SALD), a high-throughput atmospheric deposition technique that offers the advantage of producing compact and high-quality devices compared to other fabrication methods^41^. The capacitive readout is provided by a 50 nm-thick Aluminum (Al) layer, on top of the ZnO layer, coupled to a substrate Al electrode with an air gap of 2 μm (Fig. S2b). The fabrication process is described in the Supplementary Materials. Figure S2a shows a scanning electron microscope (SEM) image of a DUT measuring 50 μm long and 10 μm wide.

A biased harmonic potential difference:

$$v(t)={V}_{{\rm{DC}}}+{V}_{{\rm{AC}}}\cos (2\pi ft)$$

is applied between the top and the substrate Al electrodes, generating an electrostatic field within the air gap, which results in an attractive force between the electrodes. The magnitude of this force is proportional to *v*^2^(*t*), and it consists of a DC component as well as two time-varying components with frequencies of *f* and 2*f*. The AC voltage excites the system at a specific frequency, while the DC voltage provides a static bias that significantly influences the electrostatic force acting on the mechanical resonator. This DC bias modifies the effective spring constant of the system, inducing a static displacement in the nanocantilever and consequently shifting the resonance frequencies of the mechanical modes. By fine-tuning the DC voltage, precise alignment of the AC excitation with the target mechanical mode can be achieved, optimizing modal interactions and facilitating efficient energy transfer.

The aim of this work is to harness the out-of-plane oscillations of the beam under this force as a FC generator by incorporating it into a NEMS composed of an electrical resonator, RLC circuit, coupled to a mechanical resonator, the beam. A schematic of the NEMS is shown in Fig. S3a. We employ resonance matching^42^ to drive the NEMS. Therefore, the natural frequency of the electrical resonator *f*_*e*_ is tuned, by adjusting the circuit inductance *L*, to match either the natural frequency of the first out-of-plane bending mode (*f*_*m*_) or twice that value (2*f*_*m*_), thereby establishing a commensurate one-to-one (*f*_*e*_ ≈ *f*_*m*_) or two-to-one (*f*_*e*_ ≈ 2*f*_*m*_) ratio between an electrical and a mechanical modes, resulting in driving the 1:1 or 2:1 electromechanical modal interactions (akin to the fundamental parametric resonance or principal parametric resonance of the mechanical resonator, respectively.). The activation level and efficiency of this coupling are dependent on the combined effects of the applied AC and DC voltages. When the AC frequency matches the resonance of the electrical mode and the DC voltage is tuned to shift the target resonance frequency, optimal energy transfer is achieved. This fine tuning allows for maximum mode coupling and efficient energy exchange. In the vicinity of *f*_*e*_, the amplitude of the voltage signal supplied to the electrical resonator is amplified by approximately its quality factor^42^, enabling the system to reach the activation threshold and drive efficient electromechanical interactions.

**Fig. 1:**
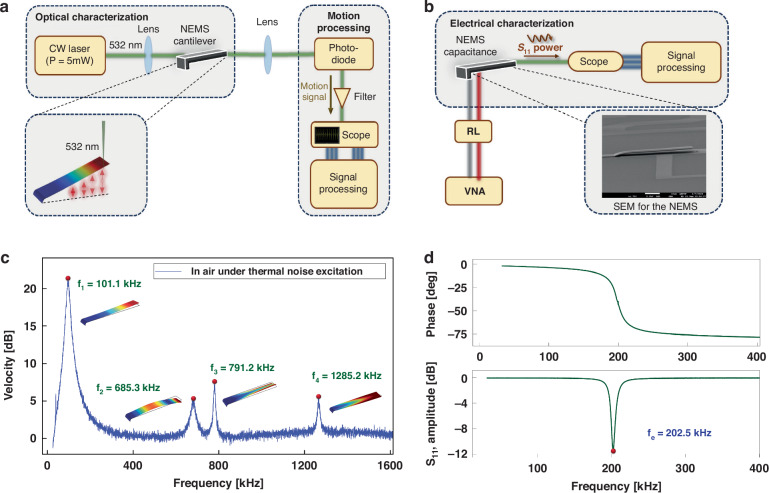
NEMS characterization. Schematics of the **a** optical and **b** electrical measurement systems used to characterize the DUT. **c** FFT of the cantilever tip velocity under thermal noise excitation. **d** The measured magnitude and phase of the returned electrical power *S*_11_ of the DUT

We employ electrical and optical measurement techniques (Fig. 1) described in the Supplementary Materials to investigate the electro-mechanical response of the NEMS. Modal analysis of the beam was first carried out via optical measurement of the tip velocity, a node-free region, under thermal noise excitation in ambient air. The Fast Fourier Transform (FFT) of the measured velocity unveiled two out-of-plane bending modes and two torsional modes (Fig. 1c). The bending modes were observed at *f*_1_ = 101.1 kHz and *f*_2_ = 685.3 kH and the torsional modes were observed at *f*_3_ = 791.2 kHz and *f*_4_ = 1285.2 kHz. The quality factors of the modes in air were evaluated using the half-power bandwidth method as *Q*_1_ = 8.5, *Q*_2_ = 18.5, *Q*_3_ = 39.5, and *Q*_4_ = 52.2.

The electrical resonance frequency was set to *f*_*e*_ = 202.5 kHz, which corresponds to 2:1 modal interaction (*f*_*e*_ ≈ 2*f*_*m*_), using a *L* = 13 mH inductor. The RLC’s overall capacitance was measured as *C* = 20 pF and a resistor (*R* = 1 k*Ω*) were introduced to prevent current overloading. The electrically measured frequency response of the NEMS in the frequency range of [10, 400] kHz is shown in Fig. 1d. The magnitude and phase plots reveal a significant drop in returned power and phase angle at *f*_*e*_. The same setup was used to drive the 1:1 modal interaction. In this case, the inductance of the electrical resonator was set to *L* = 55 mH which resulted in a measured electrical resonance of *f*_*e*_ = 101.2 kHz, corresponding to a one-to-one (*f*_*e*_ ≈ *f*_*m*_) modal interaction (Fig. S5).

### Frequency combs

#### Two-to-one electro-mechanical modal interaction

**Fig. 2:**
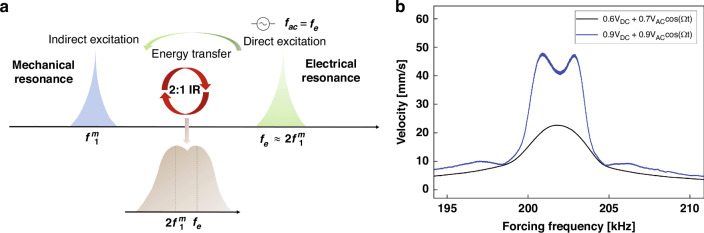
NEMS electro-mechanical modal interaction. **a** A schematic illustrating the 2:1 electro-mechanical modal interaction employed in this study. **b** The optically measured DUT frequency-response curves below (*V*_DC_ = 0.6 V, *V*_AC_ = 0.7 V) and above (*V*_DC_ = *V*_AC_ = 0.9 V) the activation level

We obtained the frequency response of the NEMS by sweeping the frequency of a biased harmonic signal in the vicinity of electrical resonance *f*_*e*_. This frequency range enables the direct excitation of the electrical mode. In the presence of quadratic coupling between the mechanical and electrical resonators, see the Supplementary Materials, and a commensurate 2:1 ratio between *f*_*e*_ and *f*_*m*_, it also results in the indirect excitation of the mechanical mode once the excitation level crosses the threshold necessary to activate the energy channel between the modes. Figure 2a provides a schematic illustration of the frequency response before and after triggering this modal interaction.

Figure 2b depicts the frequency-response curves obtained in ambient air for an excitation force below the activation level (black line) with *V*_dc_ = 0.6 V and *V*_ac_ = 0.7 V and above the activation level (blue line) with *V*_dc_ = *V*_ac_ = 0.9 V as the excitation frequency is swept from *f* = 194 kHz to 211 kHz. The frequency response transitions from a single resonant peak, corresponding to electrical resonance, below activation to the characteristic two resonant peaks above the activation level. The remarkable efficiency of this process can be seen by comparing the drive voltage used to achieve this resonance to the static pull-in voltage of the nanobeam (70 V). This highlights the FC generator’s potential for low-power and resource-efficient utilization.

**Fig. 3:**
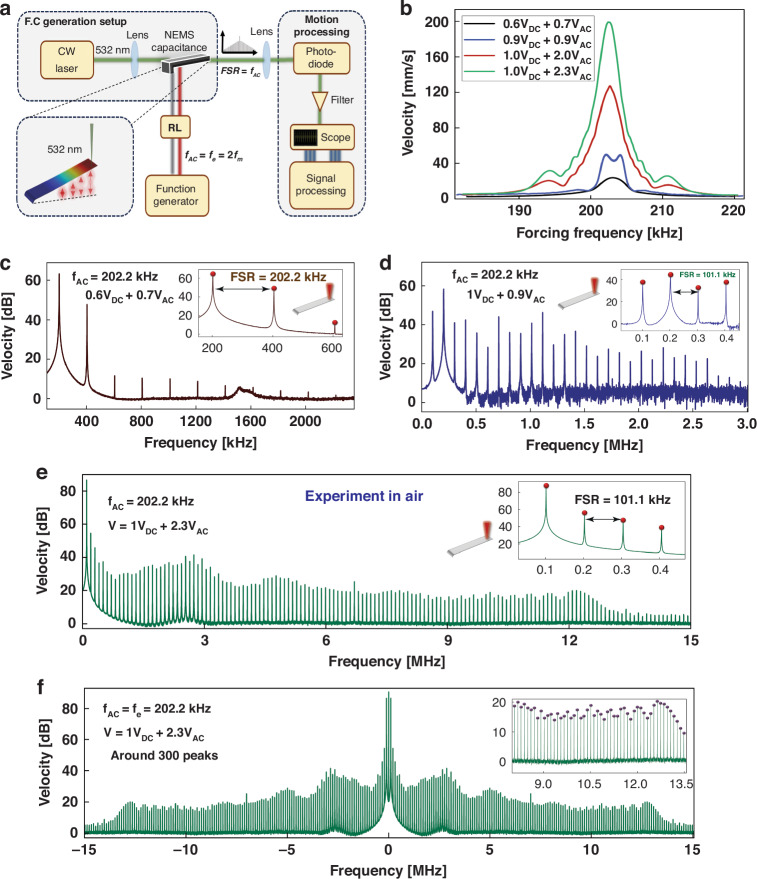
NEMS 2:1 electromechanical FCs generator. **a** A schematic of the experimental setup. **b** The measured frequency-response curves, for four levels of excitation. The measured FFT of the cantilever’s tip velocity in air **c** below, **d** just above, and **e** beyond activation level. **f** The complex FFT of the cantilever’s tip velocity in air

Increasing the excitation level with the voltage waveform *V*_DC_ = 1 V and *V*_AC_ = 2 V, the two resonant peaks merge resulting in a wider half-power bandwidth. The frequency-response curve, red line in Fig. 3b, shows a single resonant peak at *f* = 202.4 kHz, positioned between the two previous local maxima observed at 201.4 kHz and 203.2 kHz. As the forcing level increases further with a voltage waveform of *V*_DC_ = 1 V and *V*_AC_ = 2.3 V, the peak velocity experiences a significant increase, reaching tenfold of that observed below the activation level. The location of the resonant peak also shifts to lower frequencies *f* = 202.2 kHz, as larger motions result in larger effective capacitance and a stronger effective electrostatic field.

We analyzed the frequency content of the NEMS response when excited close to resonance, at a frequency of *f* = 202.2 kHz, with forcing levels below and above the activation level. The FFTs of the beam tip velocity were evaluated for the voltage waveforms (*V*_DC_ = 0.6 V, *V*_DC_ = 0.7 V), (*V*_DC_ = *V*_AC_ = 0.9 V) and (*V*_DC_ = 1 V, *V*_AC_ = 2.3 V) and are presented in Fig. 3c–e. Below the activation level (*V*_DC_ = 0.6 V, *V*_AC_ = 0.7 V), the NEMS response exhibits only a handful of uniformly spaced FCs in the spectrum up to 2 MHz corresponding to the harmonic of the excitation frequency, Free Spectral Range (*FSR*) = *f*. Just above the activation level (*V*_ac_ = *V*_DC_ = 0.9 V) (Fig. 3d), FCs appear at *f*/2 and its harmonics corresponding to the mechanical mode. The similarity in power levels across the peaks at *f*_*m*_ ≈ *f*/2 and *f*_*e*_ ≈ *f* indicate the activation of a modal interaction energy channel between the electrical and mechanical modes. Energy transfer results in a wider distribution of power over the spectrum with 30 FCs appearing in the spectrum up to 3 MHz uniformly spaced at a distance of *FSR* = *f*/2 from each other. As the driving voltage increases to *V*_DC_ = 1 V and *V*_AC_ = 2.3 V (Fig. 3e), the NEMS response evolves into a wide spectrum frequency comb with 150 electromechanical FCs, similar in power, equally spaced at *FSR* = *f*/2, and covering the frequency domain up to 15 MHz. Figure 3f shows the complex FFT of the response illustrating a broader FC centered at *f* = 0.

We compare in Table 1 our FC to other phononic FCs realized recently. The comparison underscores the significance of modal interaction in terms of power efficiency, simplicity, and the number of realized teeth across the comb, one order of magnitude higher than all others reported in the literature to date. We note that operating our frequency comb generator in a vacuum, like those reported here, will result in even wider and more powerful frequency combs.

**Table 1:** Comparison of FCs in NEMS/MEMS

Material	Pressure	Base frequency	FSR	# of combs
ZnO [this work]	Ambient	101.1 kHz	101.1 kHz	150
Si_3_N_4_^1^	<10^−5^ mbar	118 kHz	118 kHz	35
SiN^44^	<10^−4^ mbar	6.52 MHz	1.1 kHz	12
Graphene^45^	<10^−2^ mbar	2.87 MHz	28.7 kHz	16
MoS_2_^35^	2 × 10^−2^ mbar	24 MHz	26.45 kHz	15
Si^46^	High vacuum	26 kHz	0.8–2.6 kHz	25
Si^36^	70 mbar	417 kHz	0.103 kHz	27
AIN/Si^47^	–	3.86 MHz	2.6 kHz	9

The strength of the generated FCs is dependent on the activation of the modal interaction and the resulting energy transfer between the electrical and mechanical modes. To illustrate this, we excited the electrostatic NEMS with the same voltage waveform (*V*_dc_ = 1 V and *V*_ac_ = 2.3 V) at a frequency of *f* = 206 kHz, outside the interaction window. The resulting spectrum (Fig. S4) contained only 10 peaks up to 2 MHz spaced at *f*.

#### One-to-one electro-mechanical modal interaction

**Fig. 4:**
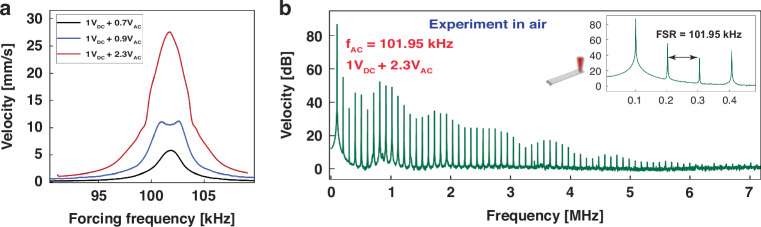
NEMS 1:1 electromechanical FCs generator. **a** The measured frequency-response curves, for three excitation levels as the frequency is swept from 90 kHz to 110 kHz. **b** The measured FFT of the cantilever’s tip velocity in air demonstrating the frequency comb generated under an excitation of V_DC_ = 1 V, V_AC_ = 2.3 V, and *f* = 101.95 kHz

In this section, we describe the generation of FCs based on 1:1 electro-mechanical modal interaction. First, the electrical resonator frequency is set to *f*_*e*_ = 101.2 kHz, such that *f*_*e*_ ≈ *f*_*m*_, as described above. The cantilever beam is then driven by a voltage waveform with a constant bias of V_DC_ = 1 V as the excitation frequency *f* is swept from 90 kHz to 110 kHz. Similar to the 2:1 case, the peak corresponding to electrical resonance is observed for low excitation levels (V_AC_ = 0.7 V), the black curve in Fig. 4a. Increasing the excitation level (V_AC_ = 0.9 V), triggers 1:1 modal interaction and results in an M-shaped frequency-response curve, blue line in Fig. 4a, where the mechanical and electrical resonance peaks are observed. Increasing the voltage amplitude further to V_AC_ = 2.3 V results in merger of the two resonant peaks into a single dominant peak between them located at *f* = 101.95 kHz.

Exciting the NEMS resonator at a frequency close to that of the peak, *f* = 101.95 kHz results in the frequency comb shown in Fig. 4b obtained from the FFT of the nano beam’s velocity. The NEMS response reveals 60 evenly spaced peaks covering the frequency spectrum from 0 to 6 MHz. The spacing between the peaks corresponds to the excitation frequency *FSR* = *f*_*e*_. Comparing these results with those obtained from the 2:1 modal interaction from the same excitation level, illustrates the weaker power interaction and resulting peaks of the 1:1 modal interaction. It is worth noting that even when the excitation levels increases, the frequency comb generated by the 1:1 modal interaction does not expand over the frequency spectrum unlike that of the 2:1 modal interaction.

### Stability of the frequency combs

#### Quality factor

In this section, we investigate frequency stability and phase coherence of the generated FCs. Frequency stability is indispensable in ensuring the precision and frequency references of wave packet generators. The frequency stability of NEMS resonators is counter proportional to the quality factor $\delta f\propto 1/\sqrt{Q}$^43^. The quality factor of the mechanical mode was measured in air at *Q*_*m*_ = 8.5. When excited within the NEMS resonator under the 1:1 modal interaction scheme, the quality factor we found to increase to *Q* = 25 which enhances the frequency stability of the NEMS generated FCs compared to the mechanical resonator. The lower impedance of the NEMS also enhances power transfer to the NEMS resulting in a 60-peak wide FC.

Under the 2:1 modal interaction excitation scheme, a substantial improvement in the quality factor, and therefore frequency stability, is realized with the measured quality factor increasing by an order-of-magnitude to *Q* = 145. This is a result of noise squeeze realized due to the principal parametric resonance excitation conditions in this case. The enhanced quality factor also expands the generated FC to cover 150 peaks. This in-depth understanding of the underlying mechanisms elucidates the pivotal role of the 2:1 and 1:1 modal interactions in enhancing the stability and range of the FCs.

#### Phase coherence

To investigate the frequency stability of the FCs, we evaluated their phase coherence. A comparison between phase coherent and phase incoherent frequency combs is shown in Fig. S6. It can be seen that phase incoherence is revealed in the time-histories of the signal (displacement) and its time derivative (velocity) which cease to have distinct maxima and minima as phase incoherence resulting in a drift between the various frequency components in the signal. Similarly, phase incoherence results in a wandering phase-space diagram randomly visiting different areas withing reachable phase-space. Unlike the case for coherent FCs where the constituent harmonics result in a single closed orbit.

**Fig. 5:**
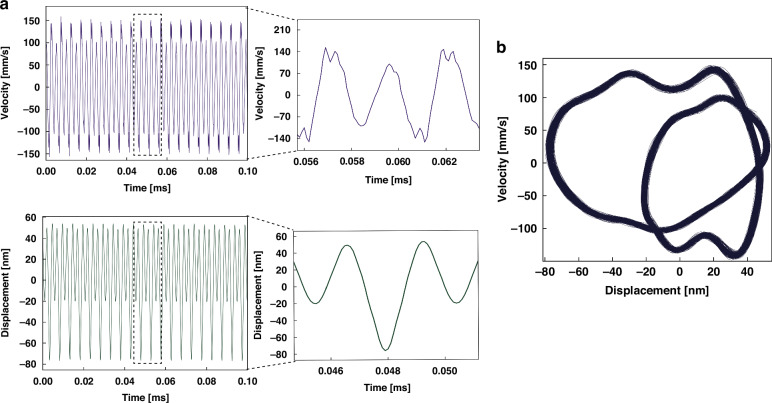
NEMS time domain response. **a** The measured time-histories of the beam tip displacement and velocity under electrical excitation with the waveform (*V*_DC_ = 1 V, *V*_AC_ = 2.3 V), and *f* = 202.2 kHz and **b** the corresponding phase-space diagram

In this work, we measured the nanobeam tip displacement and velocity in time-domain under the excitation waveform (*V*_DC_ = 1 V, *V*_AC_ = 2.3 V) and a 2:1 modal interaction with *f* = 202.2 kHz, corresponding to the FC shown in Fig. 3. The time-histories of the displacement and velocity, shown in Fig. 5a, reveals regular and distinct maxima and minima, inset of Fig. 5a, providing confirmation of the phase coherence of the FC. Likewise, the phase-space diagram of the orbit corresponding to the FC over thousands of forcing cycles (Fig. 5b) exhibit a distinct closed orbit consisting of two loops, a characteristic feature of the 2:1 modal interaction. No drift over time is observed in the phase-space diagram.

## Conclusion

In conclusion, this study has successfully demonstrated the development of an energy-efficient technique for the generation of electro-mechanical frequency comb via modal interactions between an electrical resonator and a mechanical resonator. An electrostatic NEMS made of a resonant drive circuit and an nanobeam was deployed as a platform to demonstrate the technique. In one case, the electric circuit’s inductance was tuned to match its resonant frequency to twice the resonant frequency of the beam’s out-of-plane bending mode, thus enabling an energy efficient 2:1 modal interaction between the electrical and mechanical modes. The results show the generation of 150 evenly spaced peaks covering a 15 MHz-wide spectrum, confirming successful creation of low voltage (<5 V) FCs. In another case, we used the same NEMS to generate FCs via 1:1 modal interaction by tuning the electric circuit’s inductance to match its resonant frequency to the resonant frequency of the beam’s out-of-plane bending mode. The resulting FC had 60 evenly spaced peaks covering a 6 MHz-wide spectrum. This result emphasizes the advantages of the 2:1 modal interaction in terms of frequency comb width and stability compared to the 1:1 interaction. It also demonstrates the simplicity of the fabricated nanocantilever using the AP-SALD technique and highlights the flexibility of the approach to accommodate various modal interactions.

## Supplementary information

Supplemental Material
