# Supplementary material for: NEMS generated electromechanical frequency combs
Source: Microsyst Nanoeng. 2025 Jan 15;11:8. doi: 10.1038/s41378-024-00860-9 (PMC11735631; doi:10.1038/s41378-024-00860-9)
Supplement: Supplementary file 1 — Supplemental Material [file 41378_2024_860_MOESM1_ESM.pdf]

Supplementary information for NEMS Generated Electromechanical Frequency Combs

Sasan Rahmanian[1†], Hamza Mouharrar[2*†], Rana Abdelrahman[1], Masoud Akbari[3,4,5], Yasser S. Shama[1], Kevin Musselman[3], David Muñoz-Rojas[4], Skandar Basrour[5], Eihab Abdel Rahman[1]

[1]Department of Systems Design Engineering, University of Waterloo, Waterloo, Ontario, N2L 3G1, Canada.
[2*]Renewable Energy Engineering Department, Mediterranean Institute of Technology, South Mediterranean University, Lac2, Tunis, 1053, Tunisia.
[3]Department of Mechanical and Mechatronics Engineering, University of Waterloo,Waterloo, Ontario, N2L 3G1, Canada.
[4]University Grenoble Alpes, CNRS, Grenoble INP, LMGP, 38000 Grenoble, France.
[5]University Grenoble Alpes, CNRS, Grenoble INP, TIMA, 38000 Grenoble, France.

*Corresponding author(s). E-mail(s): hamza.mouharrar@medtech.tn;
†These authors contributed equally to this work.

Fabrication process for the NEMS

The fabrication steps for the cantilever are illustrated in Figure S1. The cantilevers are fabricated on SiO2/Si wafers, starting with the patterning of aluminum on the substrate, which serves as the bottom electrode and bonding pads, using a lift-off technique (Figure S1(a)). This process involves spin-coating a negative-tone resist (ma-N 1410), performing UV lithography (λ=375 nm, $400\, mJ.cm^{-}2$), and depositing a 60 nm aluminum layer via sputtering.

Next, a 1 µm-thick sacrificial positive-tone photoresist is spin-coated on the wafer and patterned over the bottom electrode using UV lithography (λ=405 nm, $110\, mJ.cm^{-}2$) (Figure S1(b)). Following this, a 200 nm-thick ZnO layer is deposited at 200°C using Atmospheric-Pressure Spatial Atomic Layer Deposition (AP-SALD), with diethylzinc and water as the precursor and reactant, respectively, delivered by nitrogen as a carrier gas (Figure S1(c)). More details on the AP-SALD technique and deposition parameters are available in the references [1–3]. The ZnO film conforms to the substrate surface, including the sidewalls of the sacrificial photoresist, ensuring proper attachment of the structural layer to the cantilever's anchors.

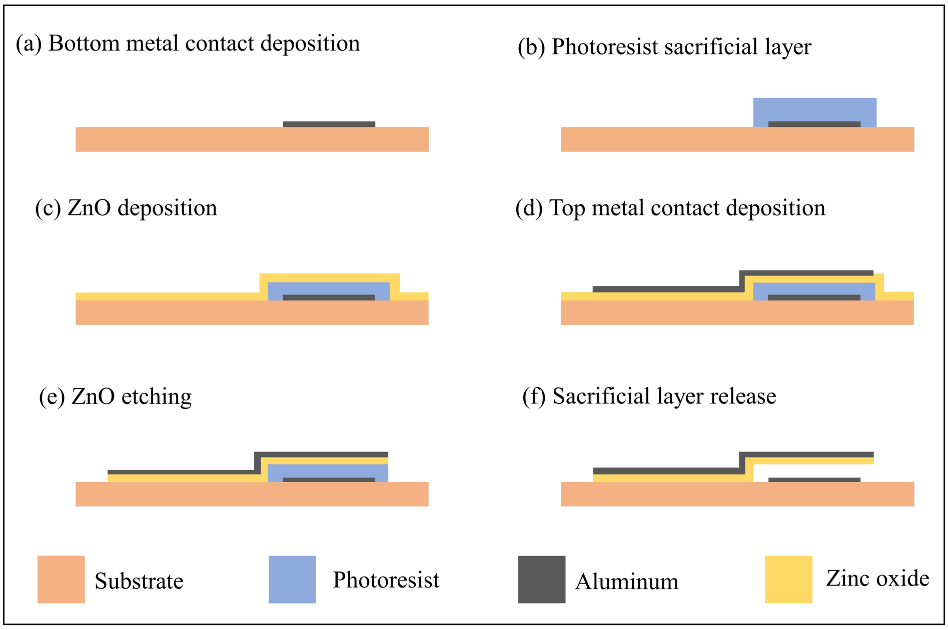

Fig. S1: Fabrication process

The top metal electrode and bonding pads are then patterned in a similar manner as the bottom electrode (Figure S1(d)). Subsequently, the ZnO layer is etched using reactive ion etching (RIE) with methane and hydrogen gases at 60°C, where the top electrode acts as both the etch mask and defines the cantilever's shape (Figure S1(e)).

Finally, the sacrificial photoresist is removed via oxygen plasma ashing ashing at 180°C (RF power of 1000 W), releasing the cantilever (Figure S1(f)). The final result is a nanocantilever beam approximately 250 nm thick, consisting of 200 nm of ZnO and 50 nm of aluminum, with a length of 50 µm and a width of 10 µm, Figure S2.

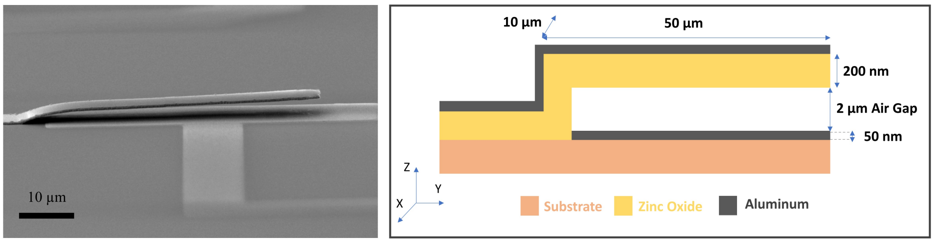

(a) (b)

Fig. S2: The NEMS cantilever; (a) an SEM image showing the beam and its anchor and (b) a schematic cross-section of the beam.

Experimental Setup

Two experimental techniques are employed to measure the electro-mechanical response of the NEMS, electrically and optically. Figure S3(a) illustrates the NEMS under study: a pair of coupled electrical, RLC circuit, and mechanical, beam, resonators. The coupling element is the electrostatic field developed between the beam and the substrate.

Electrical measurement

A Vector Network Analyzer (VNA) was employed to characterize the resonant frequency of the RLC circuit f_e. The circuit configuration is shown in Figure S3(a). The VNA supplied the input power into the NEMS and measured the returned power to evaluate the circuit's (S_{11} parameter) during a frequency sweep. The results were post-processed using a LabView software interface. This experimental setup was used for precise measurement and tuning of f_e.

Optical measurement

The displacement and velocity of the beam were measured optically using a Laser Doppler Vibrometer (LDV), Figure S3(b). In this case, the drive electrical potential difference was supplied to the NEMS via a voltage source (function generator). The laser spot was focused on the beam tip. The measured response was post-processed in the time and frequency domains to obtain the FFT and frequency response of the NEMS. This setup enables precise real-time analysis and visualization of the NEMS response. Frequency-response curves were constructed from the time-domain measurements following the procedure described by Elhady et al. [4]. The FFT plots depicting

frequency combs, we have removed the noise floor to provide a clearer view of the well-defined and discernible overall frequency comb pattern. It is worth noting that transitioning from the electrical measurement setup to the optical setup is accompanied by a decrease in the inductance of the RLC circuit. This results in a minor increase in the electrical resonant frequency.

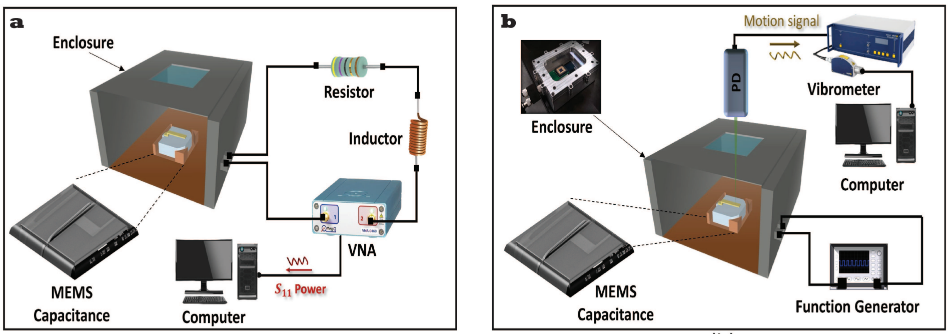

Fig. S3: The experimental setups used for (b) electrical characterization of the DUT and (b) optical characterization of the DUT as well as observation of the generated frequency combs.

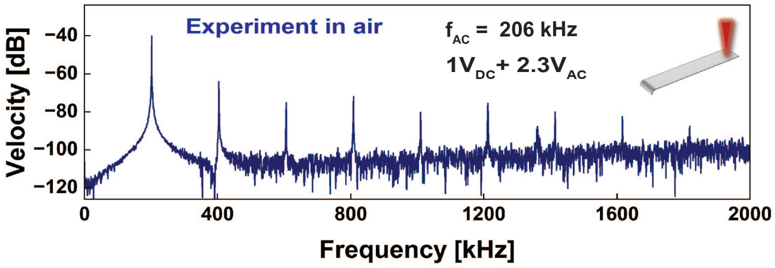

Fig. S4: The measured FFT of the cantilever's tip velocity in air under an excitation of V_{DC}=1 V, V_{AC}=2.3 V, and f = 206 kHz

Mathematical model

This section presents a mathematical model capturing the nonlinear dynamics of the NEMS. It couples a mechanical resonator to an electrical resonator. The equation of motion describing the transverse displacement of a point along the neutral axis at a distance of x from the left support of the nanobeam $w(x;t)$ can be written as follows

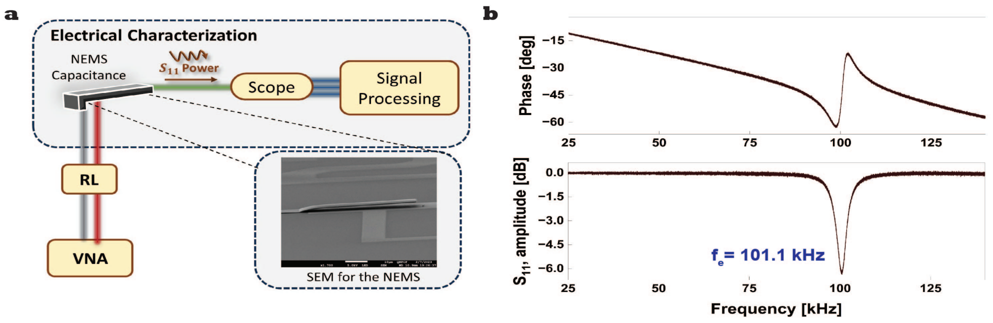

Fig. S5: Schematic of the electrical measurement system used to characterize the DUT, (b) The measured magnitude and phase of the returned electrical power S_{11} of the DUT for the 1:1 modal interaction case

[5, 6] :

$$\rho A \frac{\partial^2 w}{\partial t^2} + c_v \frac{\partial w}{\partial t} + EI \frac{\partial^4 w}{\partial x^4} = \frac{\epsilon b}{2} (\frac{q}{C_m})^2 \frac{1}{(g_0 - w)^2} \tag{ES.1}$$

subject to the boundary conditions:

$$\begin{aligned} w(0) = w'(0) = 0 \qquad & \text{at} x = 0 \\ \frac{\partial^2 w}{\partial x^2} = \frac{\partial^3 w}{\partial x^3} = 0 \qquad & \text{at} x = L \end{aligned} \tag{ES.2}$$

where L, b, and h denote the beam's length, width, and thickness, respectively, g_0 is the initial gap between the beam and the stationary electrode, and ϵ is air permittivity. The Young's modulus and density of the beam are denoted E and ρ, respectively. Its second moment of area can be found as $I = \frac{1}{12} b h^3$. Energy losses due to interactions between the beam and its support and the surrounding air are represented by linear viscous damping with a damping coefficient of c_v. The geometrical nonlinearity of inextensibility has been ignored in Eq.(ES.1) because of the small ratio of the air gap to the beam length $(\frac{g_0}{L}^2)$. The total NEMS capacitance C_m is displacement-dependent and can be evaluated as:

$$C_m = \int_0^L \frac{\varepsilon b}{(g_0 - w)} dx \tag{ES.3}$$

The nanobeam is coupled to an electrical resonator that can be represented by an RLC circuit. The ordinary differential equation describing the flow of electrical charge $q(t)$ through it can be written as

$$L_e \ddot{q} + R_e \dot{q} + \frac{1}{C_m} q = V_{in}(t) = V_{DC} + V_{AC} \cos(\Omega t) \tag{ES.4}$$

where L_e and R_e represent the inductance and resistance of the electrical circuit, respectively. A signal generator drives the circuit with a waveform that has a bias of V_{DC}, an amplitudes of V_{AC}, and a frequency of Ω. Inspecting the system equations,

Eqs. (ES.1) and (ES.4), it can be seen that the mechanical resonator is coupled to the electrical resonator via a quadratic nonlinear term, q^2 whereas the electrical resonator is coupled to the mechanical resonator via the displacement-dependent hard nonlinearity $\frac{1}{C_m}$.

The static equilibrium of the corresponding nonlinear system (w_s, q_s) should be obtained via dropping the non-autonomous term in Eq. (ES.4), and setting the time-derivative terms equal to zero to obtain the algebraic system:

$$\begin{aligned} EI\frac{d^4 w_s}{dx^4} &= \frac{\epsilon b}{2}\left(\frac{q_s}{C_s}\right)^2 \frac{1}{(g_0 - w_s)^2} \\ q_s &= V_{\mathrm{DC}} C_s \end{aligned} \tag{ES.5}$$

where

$$C_s = \int_0^L \frac{\epsilon b}{(g_0 - w_s)} dx$$

is the beam capacitance at equilibrium. The system is solved numerically by applying Galerkin's decomposition method [5, 6]. The NEMS dynamics are then truncated around the static equilibrium (operating point) by decomposing the displacement and charge into static and dynamic components, as follows:

$$\begin{aligned} w(x;t) &= w_s(x) + w_d(x;t) \\ q(t) &= q_s + q_d(t) \end{aligned} \tag{ES.6}$$

Substituting with Eq. (ES.6) into Eq. (ES.5), we obtain:

$$\begin{aligned} &\rho A \frac{\partial^2 w_d}{\partial t^2} + c_v \frac{\partial w_d}{\partial t} + EI\left(\frac{d^4 w_s}{dx^4} + \frac{\partial^4 w_d}{\partial x^4}\right) = \\ &\underbrace{\frac{\epsilon b}{2}\left(\frac{q_s + q_d}{\int_0^L \frac{\epsilon b}{(g_0 - w_s + w_d)} dx}\right)^2 \frac{1}{(g_0 - w_s - w_d)^2}}_{I_1} \end{aligned} \tag{ES.7}$$

$$L_e \ddot{q}_d + R_e \dot{q}_d + \underbrace{\frac{1}{\int_0^L \frac{\epsilon b}{(g_0 - w_s - w_d)} dx}(q_s + q_d)}_{I_2} = V_{DC} + V_{AC}\cos(\Omega t)$$

Expanding the two fractional nonlinear terms, I_1 and I_2, in terms of nonlinear polynomials, we obtain:

$$
\begin{aligned}
I_1 =& \frac{q_s^2}{C_s^2(g_0-w_s)^2} + \frac{2q_s^2}{C_s^2(g_0-w_s)^3}w_d \\
&- \frac{2q_s^2\int_0^L \frac{1}{(g_0-w_s)^2}dx}{C_s^3(g_0-w_s)^2}w_d + \frac{2q_s}{C_s^2(g_0-w_s)^2}q_d \\
&+ \frac{q_d^2}{C_s^2(g_0-w_s)^2} - \frac{4q_s\int_0^L \frac{1}{(g_0-w_s)^2}dx}{C_s^2(g_0-w_s)^2}w_dq_d \\
&+ \frac{4q_s}{C_s^2(g_0-w_s)^3}w_dq_d + \frac{3q_s^2}{C_s^2(g_0-w_s)^4}w_d^2 \\
&+ \frac{3q^{*2}\int_0^L \frac{1}{(g_0-w_s)^2}dx}{C_s^4(g_0-w_s)^2}w_d^2 \frac{q_s^2\int_0^L \frac{2}{(g_0-w_s)^3}dx}{C_s^3(g_0-w_s)^2}w_d^2
\end{aligned}
\tag{ES.8}
$$

$$
\begin{aligned}
I_2 =& \frac{q_s}{C_s} + \frac{q_d}{C_s} - \frac{\int_0^L \frac{1}{(g-w_s)^2}dx}{C_s^2}q_sw_d \\
&+ \left(\frac{(\int_0^L \frac{1}{(g-w_s)^2}dx)^2}{C_s^3} - \frac{\int_0^L \frac{2}{(g-w_s)^3}dx}{2C_s^2}\right)q_sw_d^2 \\
&- \frac{\int_0^L \frac{1}{(g-w_s)^2}dx}{C_s^2}q_dw_d
\end{aligned}
\tag{ES.9}
$$

The quadratic coupling terms in the NEMS electrical and mechanical resonators are highlighted in blue. This analysis shows that electrostatic NEMS dynamics have built-in quadratic nonlinear coupling that can trigger 2:1 modal interaction between their electrical and mechanical resonators.

Phase coherence of the Frequency combs

We considered an oscillatory signal composed of 150 harmonics to study the impact of the non-coherent phase on time-domain and frequency-domain responses and phase-space diagram.

$$
x(t) = \sum_{n=1}^{150} X_n \cos(n\Omega t + \phi_n) \tag{ES.10}
$$

Assuming Ω=5. For the case in which ϕ_i=0 (i=1,2,...,150), the signal is phase-coherent. Figure S6(a) shows the short scope of the signal in frequency domain and figures S6(c and e) show the short scope of the signal in time domain, and phase-space diagram is shown in figure S6(g). Comparing phase-space diagram and time domain responses to the NEMS responses shown in Figure 5, it can be concluded that the NEMS displacement and velocity responses obtained from experimental measurements are phase-coherent.

However, when different phase values are assumed for each harmonic, introducing non-coherency in phase, the oscillatory signal described in Eq. (ES.10) takes on the shape depicted in Figure S6(d and f). This figure illustrates that the periodic time history of the signal includes primary peaks as well as smaller irregularly distributed peaks within a single period. Furthermore, the phase-space diagram of the non-coherent signal exhibits a random pattern. It's crucial to emphasize that discerning phase coherency cannot be determined by examining the amplitude-frequency plot of a Fast Fourier Transform (FFT), as seen in Figures S6(a) and S6(b).

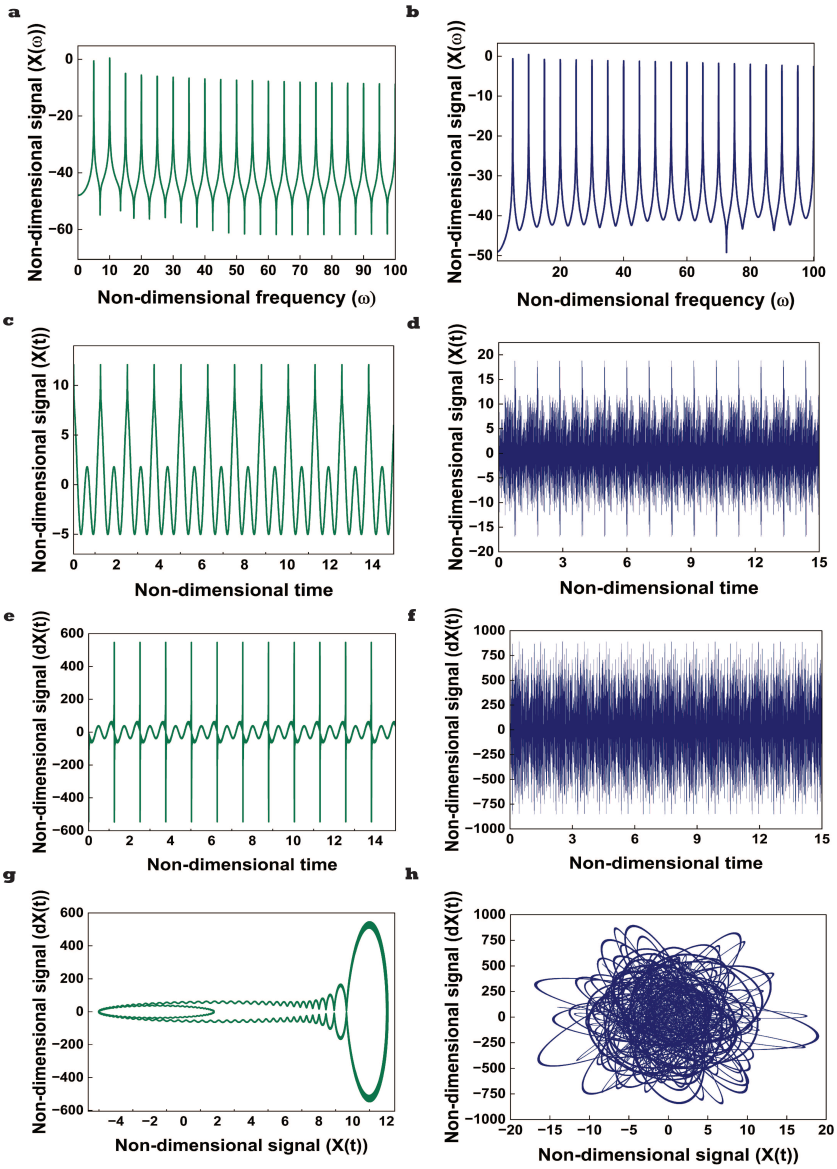

Fig. S6: The frequency-domain, the time-domain, and the phase-space diagram of the analog signal presented in Eq. (S11) are demonstrated, respectively, for $\phi_i = 0$ in (a), (c), (e) and (g), and for $\phi_i \neq 0$ ($i = 1, 2, ..., 150$) in (b), (d), (f) and (h).

References

[1] Nguyen, V.H., Resende, J., Jiménez, C., Deschanvres, J.-L., Carroy, P., Muñoz, D., Bellet, D., Muñoz-Rojas, D.: Deposition of zno based thin films by atmospheric pressure spatial atomic layer deposition for application in solar cells. Journal of Renewable and Sustainable Energy **9**(2) (2017) https://doi.org/10.1063/1.4979822

[2] Akbari, M., Mouharrar, H., Crivello, C., Defoort, M., Abdel-Rahman, E., Basrour, S., Musselman, K., Muñoz-Rojas, D.: Gas phase growth of metal-organic frameworks on microcantilevers for highly sensitive detection of volatile organic compounds. APL Materials **12**(6) (2024) https://doi.org/10.1063/5.0206295

[3] Hoye, R.L., Muñoz-Rojas, D., Musselman, K.P., Vaynzof, Y., MacManus-Driscoll, J.L.: Synthesis and modeling of uniform complex metal oxides by close-proximity atmospheric pressure chemical vapor deposition. ACS Applied Materials & Interfaces **7**(20), 10684–10694 (2015) https://doi.org/10.1021/am5073589

[4] Elhady, A., Alghamdi, M.S., Abdel-Rahman, E.: Experimental construction of force- and frequency-response curves of nonlinear resonators. Chaos: An Interdisciplinary Journal of Nonlinear Science **33**(6), 063102 (2023) https://doi.org/10.1063/5.0152209

[5] Rahmanian, S., Hosseini-Hashemi, S., Rezaei, M.: Out-of-plane motion detection in encapsulated electrostatic mems gyroscopes: Principal parametric resonance. International Journal of Mechanical Sciences **190**, 106022 (2021) https://doi.org/10.1016/j.ijmecsci.2020.106022

[6] Rahmanian, S., Hosseini-Hashemi, S., SoltanRezaee, M.: Efficient large amplitude primary resonance in in-extensional nanocapacitors: Nonlinear mean curvature component. Sci Rep **9**, 20256 (2019) https://doi.org/10.1038/s41598-019-56726-y
